# A Common *HLA-DPA1* Variant Is Associated with Hepatitis B Virus Infection but Fails to Distinguish Active from Inactive Caucasian Carriers

**DOI:** 10.1371/journal.pone.0032605

**Published:** 2012-03-20

**Authors:** Johannes Vermehren, Jörn Lötsch, Simone Susser, Sabine Wicker, Annemarie Berger, Stefan Zeuzem, Christoph Sarrazin, Alexandra Doehring

**Affiliations:** 1 pharmazentrum frankfurt/ZAFES, Institut für Klinische Pharmakologie, Klinikum der J. W. Goethe Universität, Frankfurt am Main, Germany; 2 Medizinische Klinik 1, Klinikum der J. W. Goethe Universität, Frankfurt am Main, Germany; 3 Betriebsärztlicher Dienst, Klinikum der J. W. Goethe Universität, Frankfurt am Main, Germany; 4 Institut für Medizinische Virologie, Klinikum der J. W. Goethe Universität, Frankfurt am Main, Germany; Centers for Disease Control and Prevention, United States of America

## Abstract

**Background and Aims:**

Chronic infection with the hepatitis B virus (HBV) is a major health issue worldwide. Recently, single nucleotide polymorphisms (SNPs) within the human leukocyte antigen *(HLA)-DP* locus were identified to be associated with HBV infection in Asian populations. Most significant associations were observed for the A alleles of *HLA-DPA1* rs3077 and *HLA-DPB1* rs9277535, which conferred a decreased risk for HBV infection. We assessed the implications of these variants for HBV infection in Caucasians.

**Methods:**

Two *HLA-DP* gene variants (rs3077 and rs9277535) were analyzed for associations with persistent HBV infection and with different clinical outcomes, i.e., inactive HBsAg carrier status versus progressive chronic HBV (CHB) infection in Caucasian patients (n = 201) and HBsAg negative controls (n = 235).

**Results:**

The *HLA-DPA1* rs3077 C allele was significantly associated with HBV infection (odds ratio, OR = 5.1, 95% confidence interval, CI: 1.9–13.7; p = 0.00093). However, no significant association was seen for rs3077 with progressive CHB infection versus inactive HBsAg carrier status (OR = 2.7, 95% CI: 0.6–11.1; p = 0.31). In contrast, *HLA-DPB1* rs9277535 was not associated with HBV infection in Caucasians (OR = 0.8, 95% CI: 0.4–1.9; p = 1).

**Conclusions:**

A highly significant association of *HLA-DPA1* rs3077 with HBV infection was observed in Caucasians. However, as a differentiation between different clinical courses of HBV infection was not possible, knowledge of the *HLA-DPA1* genotype cannot be translated into personalized anti-HBV therapy approaches.

## Introduction

Hepatitis B virus (HBV) infection remains a major public health concern despite the existence of a potent vaccine for more than 25 years. Worldwide, two billion people have been infected and up to 350 million people suffer from chronic HBV. They are at risk of developing liver fibrosis, cirrhosis and hepatocellular carcinoma [1]. In Germany, the prevalence of hepatitis B surface antigen (HBsAg), an indicator of current HBV infection, has been estimated to be 0.6%. Up to 40% of HBsAg carriers are reported to have a migration background from areas with higher HBV prevalence [2], [3].

Exposure to HBV results in a wide spectrum of clinical outcomes. Progression from acute to persistent HBV infection occurs in approximately 90% of individuals with perinatal infection but in only 5% or less of adults [4]. Patients with persistent HBV infection may develop chronic hepatitis B (CHB) characterized by elevated alanine aminotransferase (ALT) enzymes, high HBV DNA levels and histological lesions. In contrast, “inactive” HBsAg carriers, who generally do not need antiviral therapy, are characterized by persistently normal ALT values, low HBV DNA levels (<2000 IU/mL) and no liver injury [5], [6]. However, distinguishing between active and inactive HBV carriers can be difficult due to fluctuating ALT and HBV DNA concentrations.

Although the mechanisms of HBV persistence and factors influencing the clinical spectrum of the disease are still not fully understood, a complex interplay of viral, environmental and host factors is believed to determine the different clinical outcomes of HBV infection [4]. Moreover, family and twin studies have pointed at the important contribution of host genetic factors influencing the response to HBV infection [7], [8]. Recently, a genome-wide association study (GWAS) in a large cohort of Japanese HBV carriers and controls showed a significant association of persistent HBV infection with 11 single nucleotide polymorphisms (SNPs) within the *HLA-DP* (an HLA class II molecule) gene locus, including *HLA-DPA1* and *HLA-DPB1* [9]. The significant association of the two most or second-most strongly associated SNPs at each *HLA-DP* locus (rs3077 on *HLA-DPA1* and rs9277535 on *HLA-DPB1*) were reproduced in several large Japanese, Thai and Chinese cohorts [9], [10], [11], [12], [13]. Notably, the A alleles of both *HLA-DPA1* rs3077 and *HLA-DPB1* rs9277535 were significantly associated with a decreased risk for HBV infection. However, knowledge about variants within the *HLA-DP* locus in Caucasian patients with persistent HBV infection and their implications for clinical practice, including distinguishing inactive HBsAg carriers from CHB patients, is sparse. Therefore, this was assessed in the present investigation in 201 Caucasian patients with persistent HBV infection and 235 healthy controls who were negative for HBV serological markers.

## Methods

### Subjects and study design

The study followed the Declaration of Helsinki on Biomedical Research Involving Human Subjects. Approval from the Ethics Committee of the Medical Faculty of the J. W. Goethe University, Frankfurt, Germany, and written informed consent from each participating subject had been obtained. Consecutive patients with persistent HBV infection, defined as presence of HBsAg and HBV DNA for more than six months, were identified at the Hepatology Outpatient Clinics of the J. W. Goethe University Hospital in Frankfurt, Germany and the Saarland University Hospital in Homburg, Germany. The primary aim of this study was to assess the associations of two HLA-DP polymorphisms with disease severity in patients with persistent HBV infection. Therefore, detailed clinical data, including repeated ALT and HBV DNA measurements, were collected when available to differentiate between different courses of persistent HBV infection. This included low-viremic “inactive” HBsAg carrier status and active chronic HBV (CHB) infection according to current guideline definitions [5], [6]. In addition, HBV genotypes were also collected. Unrelated Caucasian subjects (n = 235), tested negative for HBsAg, anti-HBc, anti-HCV and anti-HIV antibodies at study inclusion, served as controls. All patients and controls were of Caucasian ethnicity by self-assignment.

### Genotyping assays

Genomic DNA was extracted from 200 µl whole blood using the EZ1 DNA Blood Kit on a BioRobot EZ1 workstation (Qiagen, Hilden, Germany). PCR primers for amplification of *HLA-DP* gene segments and the respective sequencing primers were designed with the PyroMark Assay Design software (version 2.0.1.15; Qiagen). The following primers were used: *HLA-DPA1* rs3077: forward primer: 5′-GTGTTGCTGCAGGTGACAAAAT-3′ and reverse primer: 5′-biotin-ACTGCTTTGAGGTAATGGATAAGG-3′, *HLA-DPB1* rs9277535: forward primer: 5′-CCCCAAATCAAGTTTAGTGCC-3′ and reverse primer: 5′-biotin-CATTCAAAAGTCCAAGCCGTAT-3′. Specificity of the primers was verified by gene alignment (http://www.ncbi.nlm.nih.gov/Blast/). PCR reactions (25 µl assay volume) were done on a Mastercycler® ep gradient s instrument (Eppendorf, Hamburg, Germany) using 2.5 µl genomic DNA mixed with 0.125 µl 10× HotStarTaq Plus DNA polymerase, 2.5 µl 10× HotStarTaq Plus PCR Buffer, 5 µl 5× Q-Solution, 0.5 µl dNTP mix (10 mmol/l each), 0.05 µl of one biotinylated and one nonbiotinylated PCR primer (100 µmol/l each) and 14.275 µl water. PCR reactions were carried out with an initial denaturation step at 95°C for 5 min, followed by 45 cycles at 95°C for 30 s, an annealing step at 51°C (rs3077) or 56°C (rs9277535) for 30 s and an elongation step at 72°C for 30 s and, finally, an elongation step at 72°C for 5 min. PCR products were evaluated by capillary electrophoresis on a QIAxel system (Qiagen).

Twenty-five µl of PCR templates were subsequently pipetted into wells containing 3 µl of streptavidin-coated sepharose beads (GE Healthcare Bio-Sciences, Uppsala, Sweden), 37 µl of Binding Buffer (10 mmol/l tris[hydroxylmethyl]-aminomethan, 2 mmol/l NaCl, 1 mmol/l EDTA, and 0.1% polyoxyethylenesorbitan monolaureate [Tween 20] at pH 7.6) and 15 µl HPLC-purified water. Incubation at room temperature for 10 min allowed the biotinylated DNA strands to form specific complexes with streptavidin-coated sepharose beads. The complexes were then purified and separated from the non-biotinylated DNA strands by suction on a PyroMark Q96 Vacuum Prep Workstation (Qiagen) before they were transferred to a PSQ 96 Plate Low (Qiagen) prefilled with 0.16 µl of 100 µmol/l sequencing primers (rs3077: 5′-TCACCTCATGTGAAAACTAC-3′ and rs9277535: 5′-TGCCTGATAGGACCC-3′) and 39.84 µl annealing buffer (20 mmol/l tris[hydroxymethyl]-aminomethan and 2 mmol/l magnesium acetate tetrahydrate at pH 7.6). After heating the plate to 80°C for 2 min in a PSQ 96 Sample Prep Thermoplate Low (Qiagen) it was cooled to room temperature and subsequently transferred to a PSQ® 96MA System (Qiagen) for sequencing analyses. Sequencing was performed using Pyrosequencing-specific enzyme mix, substrate mix and nucleotides (PyroMark Gold Q96 reagent set for SNP genotyping and mutation analysis; Qiagen). Two samples of each genotype and SNP were conventionally sequenced by an external laboratory (LGC Genomics, Berlin, Germany) as positive controls. Please note that in the present study the rs3077 T/C alleles correspond to the A/G alleles on the reverse strand reported previously [9], [11], and contrary to the reported studies from Asian populations, the T allele was the major allele according to the frequency in Caucasians.

### Statistics

The correspondence between the observed numbers of homozygous and heterozygous subjects with those expected from the Hardy-Weinberg equilibrium was checked by means of χ^2^ goodness-of-fit tests. A possible haploblock was identified using the three methods implemented in the Haploview software (version 4.2 [14]), i.e., 95% confidence bounds on D′ [15], four gamete rule [16] and Solid Spine of linkage disequilibrium (LD). LD was analyzed by calculating parameters D′ and r^2^ [17], [18]. Allelic frequencies of the SNPs were compared between (i) HBV infected patients and healthy controls and (ii) CHB patients and inactive HBsAg carriers using χ^2^ statistics (with correction of the alpha level of 0.05 for multiple testing according to Bonferroni; SVS 7.5.4 for Linux, Golden Helix, Bozeman, MT, USA). The dominant and recessive hereditary models were applied, i.e., comparing the number of wild-type subjects with the number of carriers of minor alleles or comparing the number of homozygous carriers of minor alleles with the rest of the subjects between the selected subcohorts, respectively. The role of the viral genotype for HBV disease activity was assessed by submitting the CHB or inactive HBsAg carrier status to binary logistic regression analysis (SPSS 20 for Linux, IBM SPSS Inc., Chicago, USA), with stepwise inclusion of candidate factors rs3077, rs9277535, and HBV genotype.

## Results

Genotypes with respect to *HLA-DPA1* rs3077 and *HLA-DPB1* rs9277535 were available from 201 patients (128 men, mean age 42±14 years) with persistent HBV infection and from 235 healthy controls with negative HBV serological markers. The minor alleles of rs3077 (C allele) and rs9277535 (G allele) were observed at frequencies of 24.1% and 25.9% in HBV patients, respectively, and in 17.8% and 24.3% of healthy controls. The two genes are located on chromosome 6 and the SNPs are 21,839 nucleotides apart according to the reference sequence given in the dbSNP database (http://www.ncbi.nlm.nih.gov/SNP/, accessed on January 14, 2012). For this distance, the SNPs are most likely not united on a haploblock and their linkage disequilibrium was weak with values of D′ = 0.44 and r^2^ = 0.18 in HBV patients and D′ = 0.43 and r^2^ = 0.13 in healthy controls.

Carriage of the minor *HLA-DPA1* rs3077 C allele was significantly associated with HBV infection (OR = 5.1, 95% confidence interval versus controls, CI: 1.9–13.7; p = 0.00093, α corrected) in the recessive hereditary model, which provided the highest significance level. A significant association was not found with the dominant model (Table 1). Notably, in the HBV patients' group there was a shift from heterozygous to homozygous carriers. That is, while 90 heterozygous and 14 homozygous carriers of the minor allele were expected from the Hardy-Weinberg equilibrium, 63 heterozygous and 28 homozygous were observed (p = 0.000012). In contrast, the distribution of rs3077 allele carriers agreed with the Hardy-Weinberg law in the healthy controls (p = 0.88). Finally, *HLA-DPB1* rs9277535 was not associated with HBV infection (p = 1; Table 1) while in both sub-cohorts, the Hardy-Weinberg equilibrium with respect to rs9277535 was observed (p = 0.98 and 0.83 for patients and controls, respectively).

**Table 1:** Association of *HLA-DPA1* variant rs3077 and *HLA-DPB1* variant rs9277535 with HBV infection.

rs3077	Cases, n (%)			Controls, n (%)*			Recessive model		Dominant model	
Phenotype**	TT	TC	CC	TT	TC	CC	OR (95% CI)	*p*	OR (95% CI)	*p*
HBV infection vs. healthy controls	125	55	21	148	69	5	5.1 (1.9–13.7)	**0.00093**	1.2 (0.8–1.8)	0.67
	0.62	0.27	0.10	0.67	0.31	0.02				
CHB infection vs. inactive HBsAg carriers (controls)	31	8	7	30	15	3	2.7 (0.6–11.1)	0.31	0.8 (0.3–1.9)	1
	0.67	0.17	0.15	0.63	0.31	0.06				

rs9277535										
Phenotype	AA	AG	GG	AA	AG	GG	OR (95% CI)	*p*	OR (95% CI)	*p*
HBV infection vs. healthy controls	110	78	13	134	88	13	1.2 (0.5–2.6)	1	1.1 (0.8–1.6)	1
	0.55	0.39	0.06	0.57	0.37	0.06				
CHB infection vs. inactive HBsAg carriers (controls)	26	18	2	29	18	1	2.1 (0.2–24.4)	1	1.2 (0.5–2.7)	1
	0.57	0.39	0.04	0.60	0.38	0.02				

* controls include either healthy subjects with negative serological HBV markers (vs. HBV patients) or inactive HBsAg carriers in the subgroup analysis of patients with HBV infection (vs. active CHB patients);

** to sustain the conventional gene description from the 5′ end to the 3′ end we changed the SNP denomination in comparison to previous publications. That is, the rs3077T/C alleles in our study correspond to the A/G alleles, respectively, in previously published studies [9], [11].

Detailed longitudinal HBV DNA and ALT concentrations were available from 94 HBV patients (Table 2). Of these, 48 (51%) were considered inactive HBsAg carriers and 46 (49%) were CHB patients. No significant association of the *HLA-DPA1* and *HLA-DPB1* variants with HBV activity was observed (p = 1; Table 1). While all but five patients had either HBV genotype A or D, neither the viral nor the host genotypes significantly influenced the viral activity and logistic regression of the activity status included none of the candidate factors.

**Table 2:** Clinical characteristics of patients with persistent HBV infection (n = 201).

	All patients	IC	CHB
Mean age ± SD in years	42±14	42±13	46±16
Male gender, n (%)	128 (64)	21 (44)	36 (78)
BMI (kg/m^2^), mean ± SD	26±5	26±5	26±5
ALT, mean ± SD	66±101	26±10	131±152
HBV-DNA, mean ± SD log_10_ IU/ml	4.4±2.8	2.0±1.0	6.8±2.4
HBV-Genotype*			
A, n (%)	20 (30)	6 (24)	7 (39)
D, n (%)	42 (63)	18 (72)	8 (44)

Characteristics are shown for all patients as well as inactive HBsAg carriers (n = 48) and active CHB patients (n = 46) in whom longitudinal data were available.

IC, inactive HBsAg carriers; CHB, active chronic hepatitis B;

* HBV genotype was available in 67 patients (25 in IC and 18 in CHB).

## Discussion

The outcome of HBV infection greatly depends on the patient's immune response at the time of exposure to the virus [4]. The underlying regulatory mechanisms of the anti-HBV immune response are still not understood in full detail. Clinical studies have shown that in subjects who achieve spontaneous clearance from acute HBV infection, a strong T cell response involving both HLA class II-restricted CD4+ helper T cells and HLA class I-restricted CD8+ cytotoxic T cells was present [4], [19]. Indeed, antigen presentation to CD4+ T cells by HLA class II molecules is believed to be crucial for the host's immune response against HBV infection [20]. This T cell response seems to be significantly attenuated in patients who develop chronic HBV infection.

HLA class II molecules are classified in three isotypes, including HLA-DR, HLA-DQ and HLA-DP. HLA-DPs are highly polymorphic and functional analyses suggest a key role for HLA-DPs in T cell allorecognition and peptide binding [21]. Therefore, polymorphisms in the *HLA-DP* gene locus may directly influence the HLA class II-mediated immune response to HBV infection. Consequently, *HLA-DP* gene polymorphisms may not only be implicated in the development of HBV persistence but may also be involved in determining different clinical outcomes of HBV infection, including active chronic HBV infection and low viremic “inactive” HBsAg carrier status. In a genome-wide association study, rs3077 and rs9277535 had been identified as the SNPs with the highest significance level of an association with HBV infection [9]. More recently, rs3077 and rs9277535 were reported to be strongly associated with decreased mRNA expression of *HLA-DPA1* and *HLA-DPB1*, respectively. Thus, lower expression of *HLA-DPA1* and *HLA-DPB1* may be associated with increased risk of chronic HBV infection [22]. Furthermore, the exchange of the *HLA-DPB1* rs9277535 wild type allele A by the minor G allele may create a new CpG methylation site. However, a functional consequence is not likely as this SNP is located in the 3′ UTR and as an *in-silico* analysis [23] did not identify a CpG island in the region surrounding the rs9277535 polymorphism.

As the first studies and reproductions were performed in Asian HBV patients only [10], [11], [12], [13], a first finding of the present study was that a highly significant association between a *HLA-DPA1* rs3077 variant allele and HBV infection was also observed in Caucasians. Interestingly, the C allele was the minor allele in our study cohort and it had an allelic frequency of 18% in healthy controls in the present study. Our observation was supported by published European HapMap cohorts (CEU population) that show C-allele frequencies of 12–16% (http://hapmap.ncbi.nlm.nih.gov/). Its higher frequency in HBV-infected patients vs. healthy controls was also supported by the HapMap CEU frequency that lay outside the binomial confidence interval of 20–26.8% observed in the present cohort of HBV-infected patients (Table 1) and a χ^2^ goodness-of-fit test versus the largest probability of 0.16 according to the HapMap CEU cohort (p = 0.036). The association followed the recessive model, which means that carriage of the minor C allele already sufficed to alter the risk of HBV inoculation whereas in Asians the association was seen in the dominant model for the minor T allele, which was a protective factor for persistent HBV infection. Our findings differ from those previously published to the extent that carriage of the minor C allele was associated with an increased risk for HBV infection in Caucasians, while the minor T allele was a protective factor for HBV infection in Asians. Together, these different studies show that the C allele was more frequently observed in both Asian and Caucasian HBV carriers.

The major finding of the present study was that the highly significant association with HBV infection did not extend to an association with the progression and activity of HBV infection. That is, no differences in allelic frequencies were observed between patients with more progressive CHB infection and those with inactive HBsAg carrier status. Likewise, rs3077 did not show a significant association with HBV-related cirrhosis or hepatocellular carcinoma in previously published reports [10], [12]. However, one limitation of our study is the relatively small sample size of each HBV patient group. Therefore, our findings need to be confirmed in larger, multi-national patient cohorts.

Our findings disappointed expectations of a clinical utility of *HLA-DP* genotyping information for decisions regarding monitoring and therapy initiation in patients with persistent HBV infection, which was the primary aim of this study. Ideally, such information could provide predictive information about whether the infection can be expected to remain stable with minimal or no liver damage or whether it progresses to active CHB and liver damage needs to be prevented by therapy initiation. Considering the small sample size, the present results do not completely exclude an association of HLA-DP genetics with the progress of HBV infection; however, the present results provide no bases that a major clinical relevance for therapy decisions for the single HBV patient will be obtained.

A third finding was that the association of rs9277535 in *HLA-DPB1* could not be reproduced in Caucasian HBV patients. Importantly, the minor G allele frequency was not rare in controls (24%) and similar frequencies have been reported from European HapMap cohorts (14%–30%). This reflects the previously observed stronger association of rs9277535 with HBV infection in Japanese as compared with Chinese. Both SNPs are found in the 3′ untranslated region of *HLA-DPA1* and *HLA-DPB1*, respectively and no functional effects for these two SNPs have been reported so far. Due to their distant location, it is highly unlikely that these polymorphisms lead to a changed amino acid sequence that directly affects the antigen-binding site. The SNPs may serve as proxy markers for closer, yet to be identified functional *HLA-DP* polymorphisms. To exclude that the negative finding was due to a different haploblock structure in Caucasians than in Asians, we analyzed several more SNPs in both genes (*HLA-DPA1* rs2301220, *HLA-DPB1* rs rs3135021, rs3128917, rs3117222, rs9380343) and found that the haploblock structure [15], separately for each gene, agreed with that reported from Asians (details not shown).

Reasons for non-agreement with the Hardy-Weinberg equilibrium include selection, familial relationship, assay error and accidental violations [24]. While the last one can never be excluded, familial relationship did not apply as the patients had not been related with each other, and assay error is unlikely since the Hardy-Weinberg equilibrium was observed in the control group and careful validation with the inclusion of a positive control sample for each genotype reduced the possible assay error. Therefore, the most likely reason for the violation of the Hardy-Weinberg law is that the immune response is greatly diminished in homozygous carriers who are therefore a selected population among carriers of persistent HBV infection.

The present results showed a highly significant association of a common variant in the *HLA-DPA1* locus with HBV infection in European patients of Caucasian ethnicity, extending the association from Asians. However, this finding has currently no major clinical implications, as differentiation between clinical courses of HBV infection is not possible and therefore, knowledge of the *HLA-DPA1* genotype cannot be translated into personalized anti-HBV therapy approaches in Caucasians.
